# Methods in organoids: a model that goes beyond our imagination

**DOI:** 10.1038/s12276-021-00685-w

**Published:** 2021-10-18

**Authors:** Bon-Kyoung Koo

**Affiliations:** Institute of Molecular Biotechnology of the Austrian Academy of Sciences (IMBA), Vienna BioCenter (VBC), Dr. Bohr-Gasse 3, 1030 Vienna, Austria

**Keywords:** Adult stem cells, Adult stem cells

Over the past decade, adult stem cell-derived organoid systems have extended into diverse fields of biomedical science: the applications range from use as a model system for the basic biology of internal organs to a new platform for human biology, enabling the live biobanking of patient samples of cancer and other diseases, genetic engineering, image-based screening, genomics, and studies of infection biology^1^. Despite the rapid progress of the field, organoid technology has thus far been utilized only by a few leading laboratories and early adopters. It often takes years of trial and error to set up, since the cultures depend on complex growth factor cocktails, the handling of organoids needs extensive training, and obtaining human samples requires ethical permissions and collaboration with clinicians. More importantly, the potential of the system will not be fully realized while unpublished know-how and trade secrets form a barrier for newcomers to the field. In this special issue, a group of expert teams have therefore made a joint effort to share the methodological details of various organoid applications.

First, Perrone et al. provide a comprehensive review of the biobanking of patient-derived gut organoids^2^. Such biobanks provide numerous new opportunities for research, but the generation of a living organoid biobank requires optimization at many steps. Based on their own experience, the authors explore all the issues that need to be considered, as well as the necessary resources and expertise to establish a fully functional organoid biobank. Next, Seidlitz et al. provide a detailed review of cancer organoid biobanking, focusing on gastrointestinal cancer^3^. This review focuses not only on the general culture methods in cancer organoid biobanking but also on the various applications of organoids for cancer studies, i.e., molecular profiling, drug screening, personalized medicine, tumor evolution, tumor microenvironment, and cancer modeling by genetic alterations. Human organoid models also provide a suitable host platform for various pathogens, which has opened up revolutionary new approaches in infection biology. Aguilar et al. guide us through the utility of organoids for infection biology and the methodological details of various applications^4^, which are further complicated by the need to coculture pathogens and host organoids. The review explains the pros and cons of different infection methods, e.g., microinjection, 2D infection, and infection on organoid chips.

Owing to the advances in organoid technology, other biotechnologies have also been applied to organoids to expand their potential. These include genetic engineering, image-based screening, and genomics. Menche et al. provide a concise guide to the available strategies for the genetic manipulation of organoids, such as viral transgenesis, CRISPR/Cas9-based gene editing, and genetic screening^5^. The authors also explore many examples and details that need to be considered when applying various genetic manipulation methods in organoids. Lukonin et al. present another valuable review highlighting their recent breakthroughs in image-based screening in organoids^6^. They also provide many points that have to be considered to achieve the most efficient screening strategy for organoid cultures. Next, Youk et al. discuss how the clonal organoid culture technique enabled single-cell genomics^7^. They compare three different single-genome amplification methods that utilize molecular amplification, the tissue’s own units, or a clonal culture of primary cells and explain what makes the adult stem cell-based organoid system the method of choice for highly accurate single-genome information. This particular review demonstrates how two seemingly unrelated fields can generate unexpected synergies that lead to a breakthrough. Finally, Peng et al. offer a comprehensive review of hepatocyte organoids and their potential future applications^8^. They show the reader how to apply existing organoid technology to a new organ system and how the knowledge gained from organ development and regeneration can be used to develop novel organoid culture models.

With this series of reviews, I wish to invite more people to join the organoid community and to adopt this state-of-the-art technology to explore their own biological questions.
